# Ultrasound-triggered with ROS-responsive SN38 nanoparticle for enhanced combination cancer immunotherapy

**DOI:** 10.3389/fimmu.2024.1339380

**Published:** 2024-03-20

**Authors:** Hongyu Liu, Yunpeng Shi, Guofeng Ji, Jukun Wang, Baodong Gai

**Affiliations:** ^1^ Department of Hepatobiliary and Pancreatic Surgery, China-Japan Union Hospital of Jilin University, Changchun, China; ^2^ Department of General Surgery, Xuanwu Hospital, Capital Medical University, Beijing, China; ^3^ Department of Gastrointestinal Colorectal and Anal Surgery, China-Japan Union of Jilin University, Changchun, China

**Keywords:** ultrasound, ROS, nanoparticle, SN38, colorectal cancer

## Abstract

Controlled generation of cytotoxic reactive oxygen species (ROS) is essential in cancer therapy. Ultrasound (US)-triggered sonodynamic therapy (SDT) has shown considerable ability to trigger *in situ* ROS generation. Unfortunately, US therapy alone is insufficient to trigger an efficient anticancer response, owing to the induction of multiple immunosuppressive factors. It was identified that 7-ethyl-10-hydroxycamptothecin (SN38) could notably inhibit DNA topoisomerase I, induce DNA damage and boost robust anticancer immunity. However, limited by the low metabolic stability, poor bioavailability, and dose-limiting toxicity, the direct usage of SN38 is inadequate in immune motivation, which limits its clinical application. Hence, new strategies are needed to improve drug delivery efficiency to enhance DNA topoisomerase I inhibition and DNA damage and elicit a vigorous anticancer cancer immunity response. Considering US irradiation can efficiently generate large amounts of ROS under low-intensity irradiation, in this study, we aimed to design a polymeric, ROS-responsive SN38 nanoformulation for *in vivo* drug delivery. Upon the in-situ generation of ROS by US therapy, controlled on-demand release of SN38 occurred in tumor sites, which enhanced DNA damage, induced DC cell maturation, and boosted anticancer immunity. Our results demonstrated that a new strategy of involving the combination of a SN38 nanoformulation and US therapy could be used for cancer immunotherapy.

## 1 Introduction

In recent years, modalities for physical therapy, such as radiosurgery, photodynamic therapy, and sonodynamic therapy (SDT), have shown impressive therapeutic effects in inhibiting tumor growth (1). Owing to the advantages of non-invasive and deep tissue-penetrating capability, ultrasound (US)-triggered SDT has already become highly popular methodology used for cancer treatment (2–5). The generation of cytotoxic reactive oxygen species (ROS), such as singlet oxygen (^1^O2) and hydrogen peroxide (H_2_O_2_), has been deemed as the main biological occurrence that kills cancer cells (6). Several factors contribute to the biological effects attributable to US irradiation, including free radical generation, cell membrane rupture, sonoporation, and heat generation (7). US not only triggers several antitumor immune responses, but also recruits immunosuppressive factors and induces various forms of immunosuppression (8). Thus, US therapy alone might be insufficient to trigger effective anticancer immune responses, and combination therapy strategies are urgently being pursued.

Over the past decade, cancer immunotherapy has enjoyed a significant breakthrough in the field of cancer therapy (9, 10). Representative immunotherapeutic modalities, especially checkpoint proteins such as programmed cell death 1 (PD-1) and cytotoxic T lymphocyte antigen 4 (CTLA-4) have achieved great success in some type of cancers in clinical settings (11). Unfortunately, most patients showed a poor response to immune checkpoint blockade therapy (12). The lack of cancer antigenicity and deficient cytotoxic T cells infiltration in tumors are two major limitations in patients exhibiting a poor response to cancer immunotherapy (13). A topoisomerase I inhibitor, 7-ethyl-10-hydroxycamptothecin (SN38), was identified as the top drug candidate for the stimulation of cytosol DNA transfer from tumor cells to antigen-presenting cells and induction of a robust anti-cancer immunotherapeutic response (14). However, the direct usage of SN38 is limited by its low metabolic stability, poor bioavailability, and dose-limiting toxicity, which renders its inadequate for immune response induction and limits its clinical application. Hence, new strategies are required to improve the SN38 delivery efficiency for enhance DNA damage and boosting anticancer immunity. Because of their small size, large surface area, and ideal *in vivo* kinetics (enhanced circulation times and high stability in blood circulation), nanoparticles could considerably increase the accumulation of drugs in certain regions via the enhanced permeability and retention (EPR) effect (15–17). Currently approved nano-delivery systems have greatly enhanced the therapeutic index of clinically validated chemotherapeutics. In addition, US-triggered SDT can generate ROS to induce cancer cells. Hence, the combination strategy of sonodynamic therapy triggered and ROS-triggered SN38 release may present a promising strategy for anti-cancer immunotherapy.

**Scheme 1:**
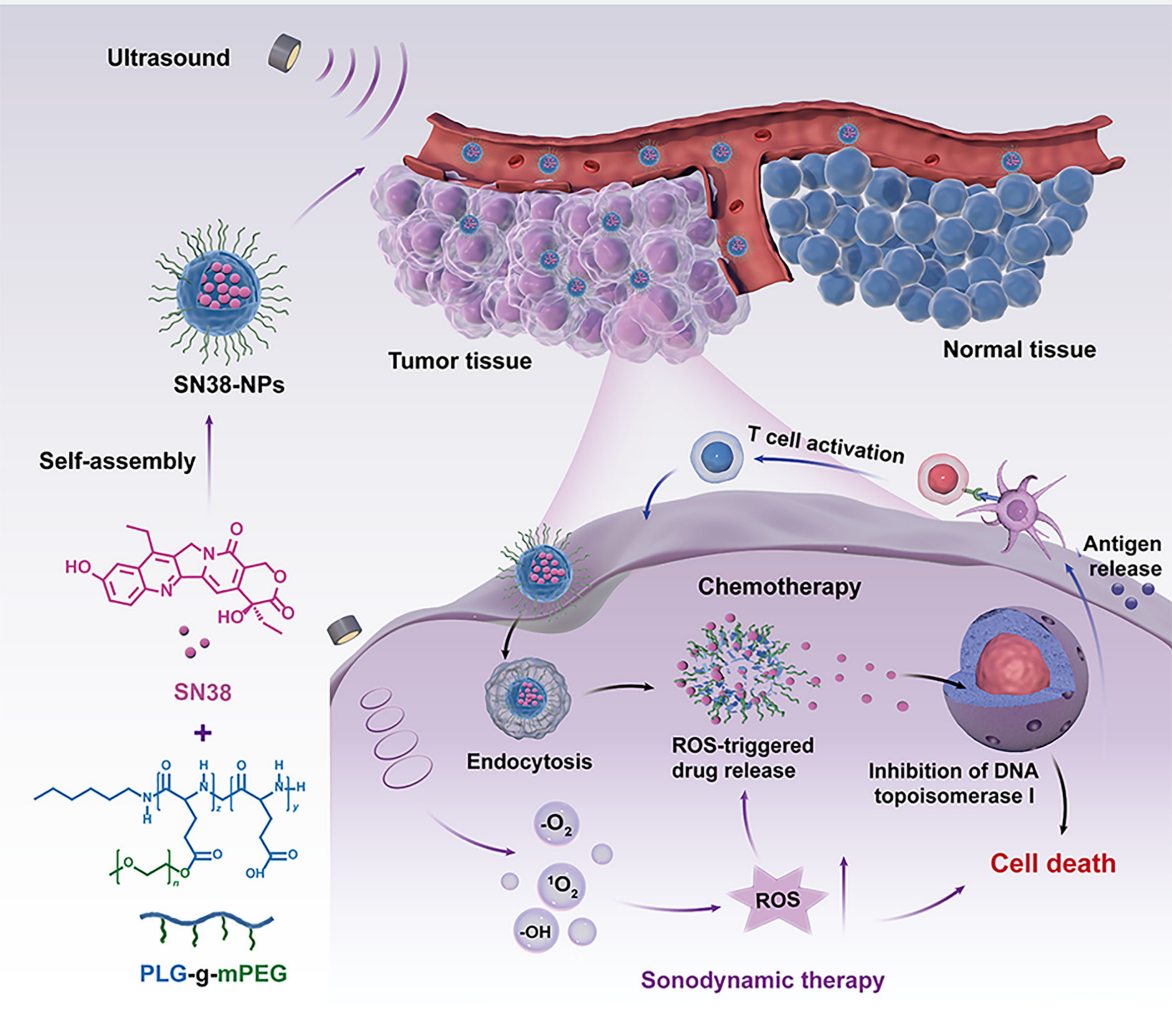
Schematic illustration of the generation of ultrasound-triggered with ROS-responsive nanoparticles for enhanced combination cancer immunotherapy. When SN38-URNPs under ultrasound conditions, the ROS-producing component generates ROS upon ultrasound irradiation, facilitating selectively release SN38 in tumor sites, eliciting robust antitumor immunity.

In this study, we screened DNA-targeting agents *in vitro* and *in vivo* and assessed if they resulted in enhanced DNA damage by SN38. Considering US irradiation can efficiently generate large amounts of ROS under low-intensity irradiation, we designed a polymeric SN38 nanoformulation with ROS-responsive for *in vivo* drug delivery. Upon the generation of ROS by US therapy, controlled on-demand release of SN38 occurred in tumor sites, which in turn enhanced DNA damage, induced DC cells maturation and activation, and boosted anticancer immunity (**Scheme 1**). Our results demonstrated a new strategy for cancer immunotherapy involving a combination of SN38 nanoformulation and US therapy.

## 2 Materials and methods

### 2.1 Materials

7-Ethyl-10-hydroxycamptothecin (SN38) was obtained from Chemical Industry (TCI) Co., Ltd (Tokyo, Japan). 3-(4,5-Dimethyl-thiazol-2-yl)-2,5-diphenyl tetrazolium bromide (MTT) was purchased from Sigma-Aldrich (Shanghai, China). Linoleic acid (LA), stearic acid (SA), trifluoroacetic acid (TFA) and triethylamine (TEA) were bought from Shanghai Rhawn Co., Ltd. (Shanghai, China). Poly (ethylene glycol) monomethyl ether (mPEG, Mn = 5000) were bought from Sigma-Aldrich (Shanghai, China). Phenyl boronic acid (PBA) was obtained from TCI Co., Ltd (Tokyo, Japan). γ-Benzyl-*_L_*-glutamate-N-carboxyanhydride (BLG-NCA) was purchased from Chengdu Enlai Biological Technology Co., Ltd. (Chengdu, China). Other reagents were purchased from the Sinopharm Chemical Reagent Co. Ltd. and used as received. RPMI 1640 and fetal bovine serum (FBS) were provided by Gibco BRL Life Technology (NY, USA). Penicillin and streptomycin were obtained from Huabei Pharmaceutical Co. Ltd. (Huabei, China). Antibodies were obtained from BD Biosciences (CA, USA) and BioLegend (CA, USA).

### 2.2 Synthesis of SN38 nanoparticles and US-induced ROS-responsive SN38 nanoparticles

Poly(*_L_*-glutamic acid)-graft-methoxy poly(ethylene glycol) copolymer (PLG-*g*-mPEG) were prepared as described previously, with minor modification (18). PLG-NH(Boc)-*g*-mPEG was obtained by esterification of PLG-*g*-mPEG with N-(Boc) ethanolamine, and then the Boc protection groups were removed by TFA, obtaining PLG-NH_2_-*g*-mPEG. The SN38-NPs and SN38-URNPs were prepared using the thin film hydration method. First, SA (28 mg) was dissolved in DMF (3 mL), and the solution was dehydrated and deoxygenated. EDC (40 mg) and NHS (30 mg) were dissolved in DMF (2 mL) and added into the SA solution for activation. PLG-NH_2_-*g*-mPEG (68 mg) and TEA (40 μL) were dissolved in DMF (6 mL) and added into the mixture. The reaction was carried out at room temperature for 24 h. The product was dialyzed in DMF and pure water and then lyophilized to generate PSA-*g*-PEG. The PLA-*g*-PEG was synthesized under the same experimental conditions except that the LA was replaced with SA. The SN38 and polymer (PLG-*g*-mPEG) were co-dissolved in CH_2_Cl_2_ at a Len-to-polymer mass ratio of 10%. The solvent was dried using a rotary evaporator at 40°C. Deionized water was added to the mixture to form micelles through self-assembly. Unassembled drug aggregates were removed via filtration through a 0.22 μm membrane and freeze-dried. SN-NPs and SN-URNPS were obtained in this way. The drug loading content (DLC) values of Len was determined using a high-performance liquid chromatography (HPLC) system, which included a Waters 2414 Refractive Index Detector, a Waters 515 HPLC pump and a reverse-phase C-18 column (Symmetry^®^).

### 2.3 Characterization

Transmission electron microscopy (TEM) images were obtained using JEOL JEM-2010 (HR). Absorption spectra were recorded using a UV-Vis spectrophotometer (Persee DU1900, Beijing, China). The hydrodynamic particle size was characterized via dynamic light scattering (DLS) using a ZetaSizer Nano-ZS90 instrument (Malvern Instrument).

### 2.4 *In vitro* drug release

The behavior of released nanoparticles was assessed under different conditions at 37°C in a release buffer (0.1 M PB buffer containing 0.5% Tween 80). SN38-NPs or URSN38-NPs (containing 0.5 mg SN38) were dissolved in 5.0 mL release buffer and transferred into a dialysis bag (MWCO 30 kDa), which was immersed into 45.0 mL of release buffer while shaking (100 rpm) the solution under the following conditions: without ultrasound (US), with US for 1 min and with US for 3 min (US: 1.0 MHz, 2.0 W/cm^2^, 50% duty cycle, 2 min). At each designed time point, 5 mL of release buffer was collected from the dialysis bag and replaced with 5 mL of fresh release buffer. The total amount of released SN38 NPs was evaluated with fluorescent spectrophotometry using a procedure consistent to that described above.

### 2.5 Cell lines and animals

Murine CT26 colon cancer cells were obtained from the BeNa Culture Collection (Beijing, China). Healthy BALB/c mice (female, 8 weeks old, 18‒20 g) and Sprague-Dawley (SD) rats (female, 7 weeks old, 200g) were bought from Beijing Vital River Laboratory Animal Technology Co., Ltd (Beijing, China). All the mice were raised separately and used according to the guidelines on Laboratory Animals of Jilin University (Jilin, China).

In order to establish a subcutaneous cancer model establishment, CT26 cells were washed with normal phosphate-buffered saline (PBS) twice, and diluted with normal PBS into a concentration of 2×10^7^ cells/mL. CT26 cells (2×10^6^ cells, 100μL) were injected into the right flank of male BALB/c mice.

### 2.6 Cytotoxicity assay *in vitro*

The cytotoxicity of SN38-URNPs was determined using the MTT assay. Typically, murine CT26 cells or 3T3 fibroblasts were seeded into 96-well plates (8000 cells per well) and cultured overnight with 200 μL RPMI 1640 or DMEM. Then, the culture medium was replaced with drug-containing fresh media. After incubation for another 48 h, 20 μL 5% MTT reagent was added into each well, followed by incubation for another 4 h. Absorbance values were determined using a microplate reader at 490 nm. The cell viability (%) was calculated as the percentage of treated cells versus untreated control cells.

### 2.7 Biodistribution study of SN38-URNPs

CT26 cells (2×10^6^ cells) were subcutaneously injected into the right flank of female BALB/c mice (8 weeks old, 18-20 g). Cy5-labeled SN38-URNPs were intravenously injected into mice when the tumor size became 300 mm^3^. At 4 h and 24 h post-injection, major organs and tumors were resected and collected for fluorescence imaging. The tissue fluorescence intensity was visualized with the Davinch-Invivo HR imaging system (Davinch, Korea) at excitation and emission wavelengths of 650 nm and 700 nm, respectively.

### 2.8 Murine CT26 subcutaneous cancer therapy

After tumor cell inoculation, mice were randomly divided into eight groups when the tumor volumes became approximately 100 mm^3^. Mice were treated with PBS, SN38 (10 mg/kg), US (1.0 MHz, 2.0 W/cm^2^, 50% duty cycle, 2 min), SN38 (10 mg/kg) +US, SN38-NPs (10 mg/kg at SN38), SN-38URNPs (10 mg/kg at SN38), SN38-NPs+US, and SN-38URNPs. Treatments were scheduled every two days, and a total of three treatments were administered. Body weights and tumor volumes were recorded every other day. The tumor volume was measured with callipers and calculated as follows: tumor volume (V) = a × b^2^/2, where a and b represent the major and minor axes of the tumor, respectively. The tumor suppression rate (TSR) was calculated as follows: TSR (%) = [(V_c_ - V_x_)/V_c_] × 100%, where V_c_ and V_x_ represent the mean tumor volumes of the PBS and treatment groups, respectively.

### 2.9 Serum biochemical parameters

Peripheral blood was collected from mice at the end of the treatment process. The concentration of alanine transaminase (ATL), aspartate transaminase (AST), and blood urea nitrogen (BUN) in the serum were determined with kits according to the manufacturer’s instructions and compared.

### 2.10 Histological analysis

Mice were euthanatized at the end of the observation period. Excised tumors were fixed in a 4% paraformaldehyde solution, embedded in paraffin, and sliced into 5 μm-thick sections. Then, slices were stained with hematoxylin and eosin (H&E) to examine their pathology. Histological photos were observed via light microscopy (IX71, Olympus, Japan).

### 2.11 Flow cytometry analysis

Tumors and spleens were harvested at the end of the experiment. The tumors were cut into small pieces and homogenized with RPMI 1640 containing collagenase IV. Then the supernatant from the digested tumor tissues were collected, filtered, centrifuged and resuspended. The spleen was mechanically ground, filtered, and resuspended in RPMI 1640. Red blood cell lysis buffer was used to lyse erythrocytes. Finally, cell suspensions were stained with fluorophore-conjugated antibodies, and flow cytometry analysis was performed using a BD FACS Celesta flow cytometer.

### 2.12 Cytokine analysis

Peripheral blood was collected from mice at the end of the treatment process. Concentrations of IL-6, IFN-γ, and TNF-α in the serum were determined with an ELISA kit according to the manufacturer’s instructions. Cytokine concentrations in the serum were presented in terms of pg/mL of protein.

### 2.13 Statistical analysis

Two-tailed unpaired Student’s t-tests were performed to compare 2 treated groups. All results were presented as mean ± S.D. values. Differences were considered significant when P< 0.05 (*P < 0.05, **P < 0.01, ***P < 0.001, ****P < 0.0001).

## 3 Results

### 3.1 Synthesis and characterization of SN38-NPs and SN38-URNPs

**Figure 1:**
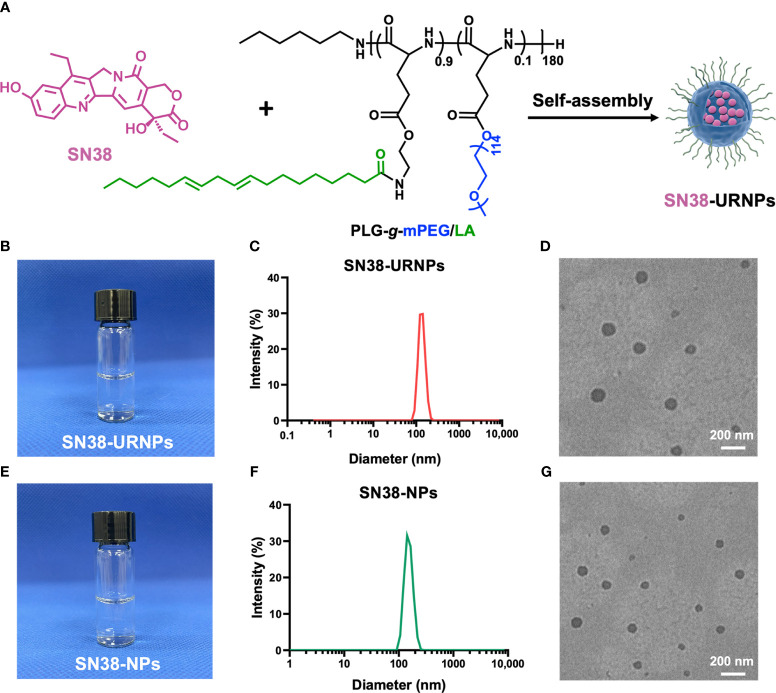
Synthesis and characterization of SN38-URNPs and SN38-NPs. **(A)** Schematic illustration of the synthesis route for SN38-URNPs. **(B–D)** Representative photo and TEM image and hydrodynamic diameter distribution as measured by DLS of SN38-URNPs. scale bar is 200 nm. **(E–G)** Representative photo and TEM image and hydrodynamic diameter distribution as measured by DLS of SN38-NPs. scale bar is 200 nm.

The nano-delivery system was demonstrated to magnify the immunogenic responses of chemotherapeutic drugs by reducing the toxicity and inhibitory effects on the immune system (19). We developed a polymeric nanoformulation of SN38 by conjugating SN38 to poly(*_L_*-glutamic acid)-*g*-methoxy poly(ethylene glycol) (PLG-*g*-mPEG) via Yamaguchi esterification for tumor-targeted therapy. PLG-*g*-mPEG was synthesized as described in a previous work (18) (**Figure 1A**). The ^1^H NMR spectrum of SN38-NPs and SN38-URNPs was shown in **Supplementary Figures 1**, **2**. The peaks at δ of 0.90 ppm was assigned to the protons of the methyl (-CH_3_, a) of 1-hexylamine. The peaks at δ of 2.83 ppm and 3.01 ppm were assigned to the protons of the methylene (-CH_2_-CH_2_-NH_2_, f) of the PLG-NH_2_-*g*-mPEG (**Supplementary Figure 1**). According to the ^1^H NMR spectrum of NPs, the peaks at δ of 5.15 ppm indicated that SA was successfully bonded to the PLG-NH_2_-*g*-mPEG, while no corresponding characteristic peak is found for non-responsive polymer materials (PLA-*g*-mPEG) (**Supplementary Figure 2**).

As shown in **Figure 1C**, DLS results showed that the prepared SN38-URNPs had uniform size distribution with an average diameter of approximately 120-140 nm. Typical TEM image indicated that spherical emulsion droplets with an average size of approximately 140 nm were formed under dehydrated conditions (**Figure 1D**), which is in accordance with the results of DLS. SN38-NPs had a uniform size distribution with an average diameter of approximately 100-120 nm (**Figures 1F, G**). These results validated the fact that SN38-NPs and SN38-URNPs with small droplet size, uniform size distribution, and good stability had been prepared effectively.

### 3.2 *In vitro* cytotoxicity

**Figure 2:**
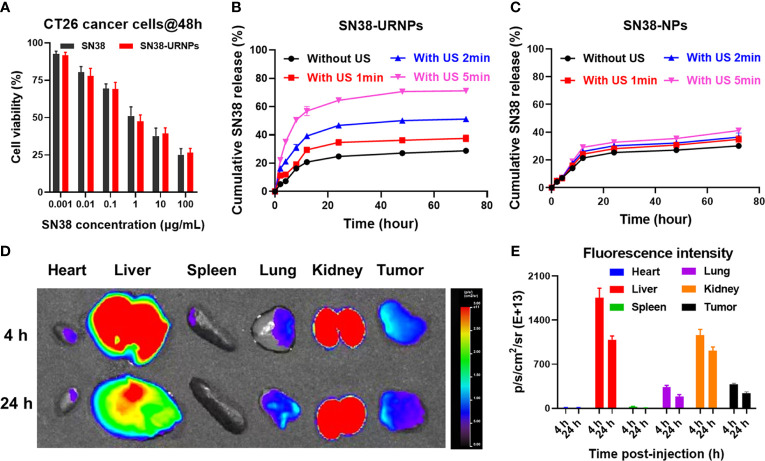
*In vitro* cell cytotoxicity and release study and *in vivo* biodistribution study of SN38-URNPs. **(A)** *In vitro* cell viability of mouse CT26 cells incubated with free SN38 and SN38-URNPs for 48 h (*n* = 3). **(B, C)** *In vitro* release profiles of SN38 from SN38-URNPs or SN38-NPs in pH 7.4 saline solutions at various simulated conditions: without ultrasound (US: 1.0 MHz, 2.0 W/cm^2^, 50% duty cycle, 2 min), with US 1min, with US 2 min and with US 5 min. (*n* = 3). **(D)** Representative fluorescence images of major organs and tumors captured at 4 h and 24 h post i.v. injection of Cy5-labeled URNPs. **(E)** Fluorescence intensity of major organs and tumors at 4 and 24 h (*n* = 3). Data are presented as means ± S.D.

The cytotoxicity of SN38-URNPs to CT26 cells was assessed in mouse CT26 tumor cells. The MTT assay was applied to CT 26 tumor cells. As shown in **Figure 2A**, the MTT assay showed that the cytotoxicity of SN38-URNPs to CT26 cells was dose- and time- dependent. At a high dosage, SN38-URNPs and free SN38 significantly decreased the rate of tumor cell proliferation at a high dosage. In addition, SN38-URNPs showed slightly lower toxicity than free SN38 in CT26 tumor cells. The strong toxicity of SN38 to tumor cells is similar to previous reports (14).

### 3.3 *In vitro* drug release of SN38-NPs and SN38-URNPs

The release of SN38 from SN38-NPs and SN38-URNPs *in vitro* was studied under different conditions (without US, with US for 1 min, with US for 2 min, with US for 5 min). As shown in **Figure 2B**, the rate of release of SN38 from SN38-URNPs increased with the increase in the time for which US was performed. In contrast, the drug release rates of SN38 from SN38-NPs showed negligible differences irrespective of US application (**Figure 2C**). The cumulative release rate of SN38 from SN38-URNPs within 48 h was 70.6%. In contrast, the cumulative release rate of SN38 from SN38-NPs or SN38-URNPs (without US) within 48 h was below 30%. The improved release rate of SN38 may be attributed to US-induced ROS generation.

### 3.4 Biodistribution study of SN38-URNPs

Cy5 labelling is widely applied in nanoparticle biodistribution studies. In this study, Cy5 was used to assess the *in vivo* biodistribution of the applied SN38-URNPs. First, CT26 cells (2×10^6^ cells) were injected subcutaneously into the right flank of female BALB/c mice (8 weeks old, 18‒20 g). Then, Cy5-labeled SN38-URNPs were intravenously injected into the mice when the tumor size became 300 mm^3^. At 4 h and 24 h post-injection, major organs and the tumor were resected and collected for fluorescence imaging. A quantitative analysis of Cy5 fluorescence intensity was also conducted to determine Cy5 distribution in different organs. As shown in **Figures 2D, E**, the liver and tumor were the major sites of SN38-URNP accumulation, and strong signals were observed in the tumor at 24 h after injection.

### 3.5 Antitumor efficacy and survival time *in vivo*

**Figure 3:**
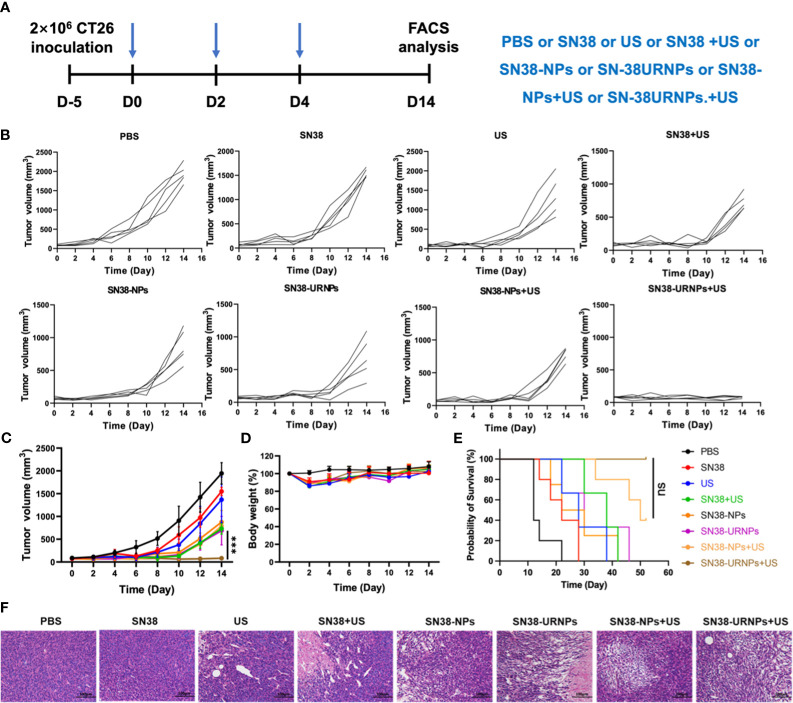
Tumor inhibition study in subcutaneous CT26 tumor model. **(A)** The therapeutic schedule of the *in vivo* study. **(B–E)** Tumor volume, body weight changes and survival curves of CT26 tumor-bearing mice after receiving various treatments (*n* = 5). **(F)** H&E staining of tumor sections after different treatments. Data are presented as means ± S.D. (***P < 0.001).

Antitumor effects were evaluated using a mouse CT26 subcutaneous tumor model where mice were administered the following different treatments: 1) PBS: without any treatment; 2) SN38 (0.1 mg/ml); 3) US (808 nm, 0.48 W/cm^2^) for 5 min; 4) SN38 +US; 5) SN38-NPs (non-ROS-responsive); 6) SN38-URNPs (ROS-responsive); 7) SN38-NPs+US; 8) SN38-URNPs+US (**Figure 3A**). Tumour volumes were monitored each day during treatment to assess the antitumor efficacy of each approach. As shown in **Figures 3B–E**, SN38 and US treatment slightly suppressed tumor growth and failed to improve the post-surgery survival rate. Tumour growth was significantly delayed in mice treated with SN38+US, SN38-NPs, SN38-URNPs, and SN38-NPs+US. Unfortunately, the therapeutic effects were not long-lasting (**Figure 3C**). As a result, the median survival time of the mice treated with SN38-NPs, SN38-URNPs, and SN38-NPs+US was not prolonged, compared to the free SN38 and US group (**Figure 3E**). Notably, sustained and thorough tumor eradication was achieved in the SN38-URNPs+US group (**Figure 3C**). At day 14 post tumor inoculation, the TSR% was 96.6% in mice treated with SN38-URNPs+US and resulted in a remarkable survival advantage, as compared to other groups (60 days). All these data confirmed that the use of SN38-URNPs plus US resulted in considerable therapeutic effects and could improve the survival time of CT26-bearing mice.

Changes in mouse body weight were monitored to assess the adverse effects of drugs. As depicted in **Figure 3D**, all treatment groups except the PBS group showed an ˜10% loss in body weight in the initial treatment stage, indicating the acute toxicity of SN38. The mice quickly regained their weight subsequently. Histological analysis was performed to evaluate the antitumor activity further. Large areas of necrosis were observed in the SN38-URNPs and US treated group, as compared to the other groups (**Figure 3F**). AST, ALT, and BUN levels were measured to assess liver and kidney functions in treated mice. As depicted in **Supplementary Figure 3**, there was no statistically significant difference in serum AST, ALT, and BUN levels compared to those in healthy BALB/c mice. The liver and kidney function analyses validated the fact that treatment with both SN38-URNPs and US did not affect liver and kidney conditions in CT26 tumor-bearing mice. These results demonstrated that the administration of SN38-URNPs or US or both was associated with a safe and tolerable side effect profile.

**Figure 4:**
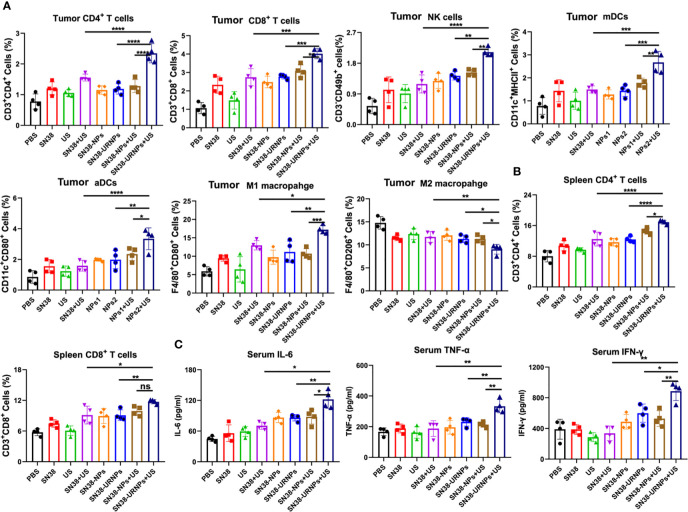
Flow cytometry analysis results of tumors and spleens after various treatments. **(A)** Tumoral CD4**^+^** T cells, CD8**^+^** T cells, NK cells, mDCs, aDCs, M1 and M2 macrophages (*n* = 4). **(B)** Spleenal CD4**^+^** and CD8**^+^** T cells. **(C)** Serum concentrations of IL-6, IFN-α, and TNF-α. (*n* = 4). Data are presented as means ± S.D. (*P < 0.05, **P < 0.01, and ***P < 0.001).

We analyzed the immune microenvironment of the tumor tissues at 14 days after various treatments. Tumors were collected from mice for flow cytometry analysis. As reported previously, SN38 could activate the STING pathway to promote DC cell maturation and activation, enhance antigen presentation ability, and finally elicit a robust T cell immune response at the tumor site (20). First, we analyzed the maturation and activation of DC cells in tumors. As expected, the proportion of matured and activated DC cells in tumor cells of mice treated with SN38-NPs or SN38-URNPs was increased significantly, and this was accompanied by elevated levels of IFN-γ, IL-6, and TNF-α in the serum (**Figures 4A, C**, and **Supplementary Figure 4**). In addition, increased numbers of infiltrating T cells were also detected, especially in the SN38-URNPs plus US treatment group, in which the highest proportions of NK cells, CD4^+^ T, and CD8^+^ T cells were detected among all the treated groups (**Figure 4A** and **Supplementary Figures 5–7**), suggesting that both innate and adaptive immune responses were generated. Numerous studies have demonstrated that tumor-associated macrophages exhibit a series of functions that promote tumor development, which include the support of the proliferation, invasion, and metastasis of tumor cells, and were highly correlated with a poor prognosis in tumor patients (21). Macrophages can switch between two main phenotypes, i.e., the anti-tumorigenic M1 phenotype and pro-tumorigenic M2 phenotype. A reduction in the M2 macrophage number or increment in the M1 macrophage number is critical for enhancing T cell antitumor immunity and inhibiting tumor growth (22). As depicted in **Figure 4A**, a considerably increased proportion of M1 macrophages and decreased proportion of M2 macrophages was detected within the tumor after treatment with SN38-URNPs plus US, indicating that the tumor immune microenvironment was reprogrammed, and immune stimulation occurred instead of immune suppression. Systemic immune response is required for effective cancer immunotherapy (22). We checked the immune cell status in spleens at the end of treatment. Increased proportions of CD4^+^ T and CD8^+^ T cells were detected in mice treated with SN38-URNPs plus US, suggesting that systemic immune had been triggered.

## 4 Discussion

Despite improvements in new drugs and therapeutic modalities, curative effect remains unsatisfactory in patients suffering from cancer, and the prognosis remains poor. Hence, novel cancer treatment strategies are still to be explored. Modalities for physical therapy, including radiotherapy, PDT, and SDT, have shown impressive therapeutic effects in inhibiting tumor growth (1). Radiation therapy involves the use of high-energy particles or waves, such as x-rays, gamma rays, electron beams, or protons, to induce cancer cell death and tumor shrinkage shrink tumors (23). However, non-contained radiation can lead to harmful effects and damages nearby healthy cells. Photodynamic therapy (PDT) can damage cancer cells by producing ROS and causing DNA damage (24). However, poor tissue penetration of PDT has limited its use to superficial lesions. SDT, which is based on PDT, has a stronger penetration ability in biological tissues compared to photons. Recent studies of US-triggered SDT have shown its effectiveness in killing tumor cells (25). However, the anticancer activity of SDT is limited to tumor eradication instead of tumor shrinkage, which may be ascribed to induces various forms of immunosuppression. Thus, US therapy alone might be insufficient to trigger effective anticancer immune responses, and combination therapy strategies are urgently being pursued.

SN38 was identified as the top drug candidate for the stimulation of cytosol DNA transfer from tumor cells to antigen-presenting cells and induction of a robust anti-cancer immunotherapeutic response, which may overcome the deficiency of US-triggered SDT. However, the direct usage of SN38 is limited by its low metabolic stability, poor bioavailability, and dose-limiting toxicity and side effects. Nanomedicines exert great promise for drug delivery to address the drawbacks of systemic administration through passive and active tumor targeting. In addition, US-triggered SDT can generate ROS to induce cancer cells. Hence, we designed a polymeric, ROS-responsive SN38 nanoformulation for *in vivo* drug delivery, based on the fact that US therapy could efficiently generate ROS.

We developed a polymeric nanoformulation of SN38 by conjugating SN38 to poly(*_L_*-glutamic acid)-g-methoxy poly(ethylene glycol) (PLG-*g*-mPEG) via Yamaguchi esterification for tumor-targeted therapy. PLG-g-mPEG was synthesized as described in a previous work (18). DLS results showed that the prepared SN38-URNPs had uniform size distribution with an average diameter of approximately 120-140 nm. Typical TEM image indicated that spherical emulsion droplets with an average size of approximately 140 nm were formed under dehydrated conditions. In this study, Cy5 was used to assess the *in vivo* biodistribution of the applied SN38-URNPs. The liver and tumor were the major sites of SN38-URNP accumulation, and strong signals were observed in the tumor at 24 h after injection.

Antitumor effects were evaluated using a mouse CT26 subcutaneous tumor model. SN38 and US treatment slightly suppressed tumor growth and failed to improve the post-surgery survival rate. Tumour growth was significantly delayed in mice treated with SN38+US, SN38-NPs, SN38-URNPs, and SN38-NPs+US. Unfortunately, the therapeutic effects were not long-lasting. As a result, the median survival time of the mice treated with SN38-NPs, SN38-URNPs, and SN38-NPs+US was not prolonged, compared to the free SN38 and US group. Notably, sustained and thorough tumor eradication was achieved in the SN38-URNPs+US group, and the TSR% was 96.6% in mice treated with SN38-URNPs+US and resulted in a remarkable survival advantage, as compared to other groups (60 days). The antitumor study confirmed that the use of SN38-URNPs plus US resulted in considerable therapeutic effects and could improve the survival time of CT26-bearing mice.

We further analyzed the immune microenvironment of the tumor tissues at 14 days after various treatments. SN38 could activate the STING pathway to promote DC cell maturation and activation, enhance antigen presentation ability, and finally elicit a robust T cell immune response at the tumor site (20). As expected, the proportion of matured and activated DC cells in tumor cells of mice treated with SN38-NPs or SN38-URNPs was increased significantly, and this was accompanied by elevated levels of IFN-γ, IL-6, and TNF-α in the serum. In addition, increased numbers of infiltrating T cells were also detected, especially in the SN38-URNPs plus US treatment group, in which the highest proportions of NK cells, CD4^+^ T, and CD8^+^ T cells were detected among all the treated groups, suggesting that both innate and adaptive immune responses were generated. In addition, a considerably increased proportion of M1 macrophages and decreased proportion of M2 macrophages was detected within the tumor after treatment with SN38-URNPs plus US, indicating that the tumor immune microenvironment was reprogrammed, and immune stimulation occurred instead of immune suppression. We also checked the immune cell status in spleens at the end of treatment. Increased proportions of CD4^+^ T and CD8^+^ T cells were detected in mice treated with SN38-URNPs plus US, suggesting that systemic immune had been triggered.

In summary, we designed a polymeric, ROS-responsive SN38 nanoformulation for *in vivo* drug delivery, since US therapy could efficiently generate ROS. Upon the generation of ROS by US therapy, controlled on-demand release of SN38 occurred in tumor sites, which in turn enhanced DNA damage, induced DC cell maturation and activation, and boosted anticancer immunity. Our results demonstrated a new anticancer immunotherapy strategy involving the combination of SN38 nanoformulation and US therapy.

## Data availability statement

The original contributions presented in the study are included in the article/**Supplementary material**. Further inquiries can be directed to the corresponding author.

## Ethics statement

The animal study was approved by the Animal Care and Committee of Jilin University. The study was conducted in accordance with the local legislation and institutional requirements.

## Author contributions

HL: Formal analysis, Writing – original draft. YS: Formal analysis, Writing – original draft. GJ: Writing – review & editing. JW: Writing – review & editing. BG: Funding acquisition, Supervision, Writing – review & editing.
