# Supplementary material for: Ultrasound-triggered with ROS-responsive SN38 nanoparticle for enhanced combination cancer immunotherapy
Source: Front Immunol. 2024 Mar 20;15:1339380. doi: 10.3389/fimmu.2024.1339380 (PMC10987707; doi:10.3389/fimmu.2024.1339380)
Supplement: Supplementary file 1 [file DataSheet_1.docx]

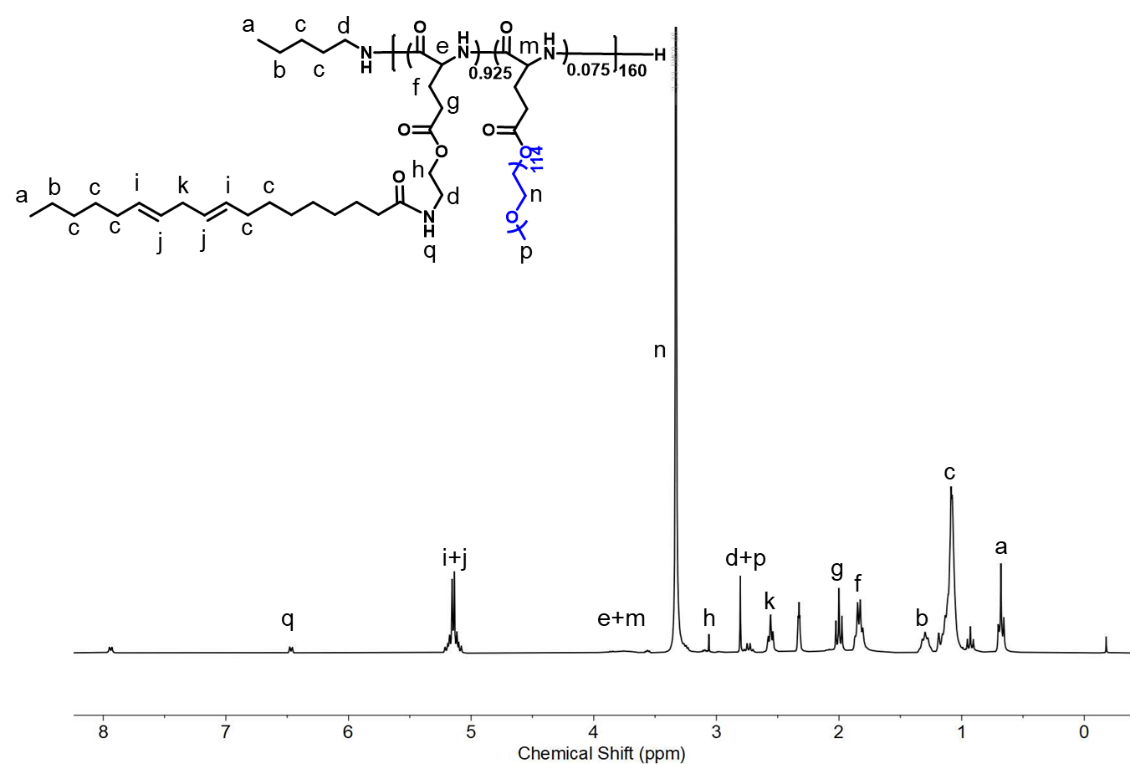


***Supplemental Figure 1*.** ^1^H NMR spectrums of SN38-URNPs in D_2_O with a drop of NaOD and DMSO-d6.


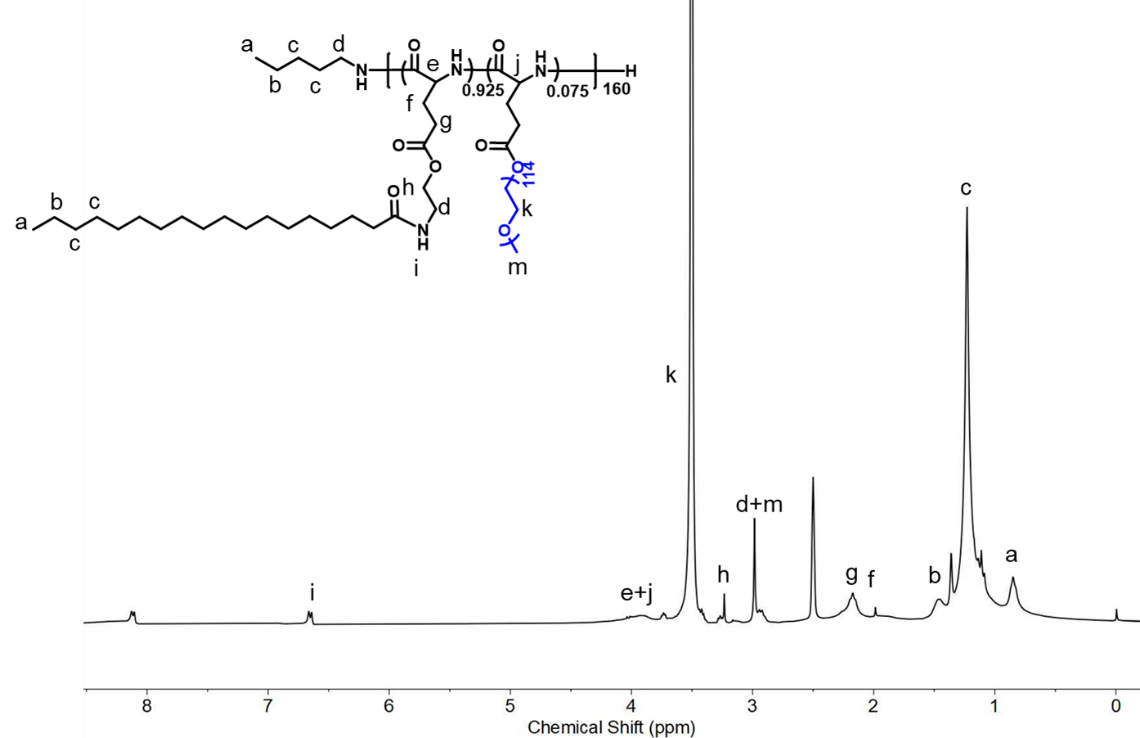


***Supplemental Figure 2*.** ^1^H NMR spectrums of SN38-NPs in D_2_O with a drop of NaOD and DMSO-d6.


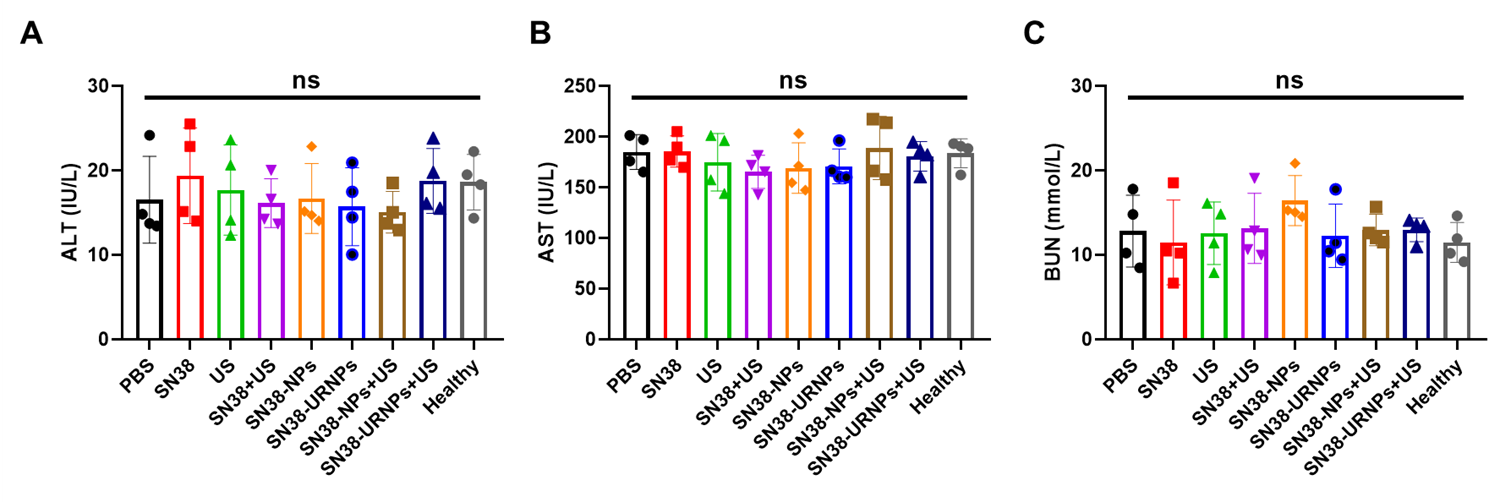


***Supplemental Figure 3*.**  Serum levels of aspartate aminotransferase (AST)**(A)**, alanine aminotransferase (ALT) **(B)**, and blood urea nitrogen (BUN) **(C)** in mice after various treatments (n = 4). Data are shown as mean ± SD from three different experiments. (ns, not significant).


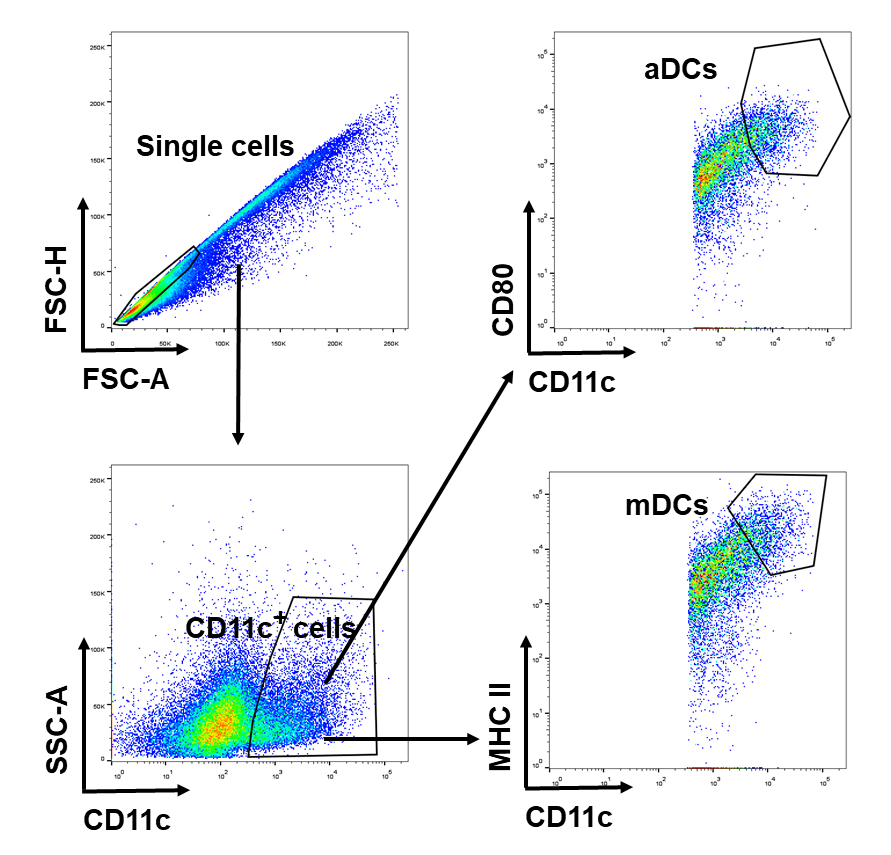


***Supplemental Figure 4*.**  Gating strategy for active DCs and matured DCs.


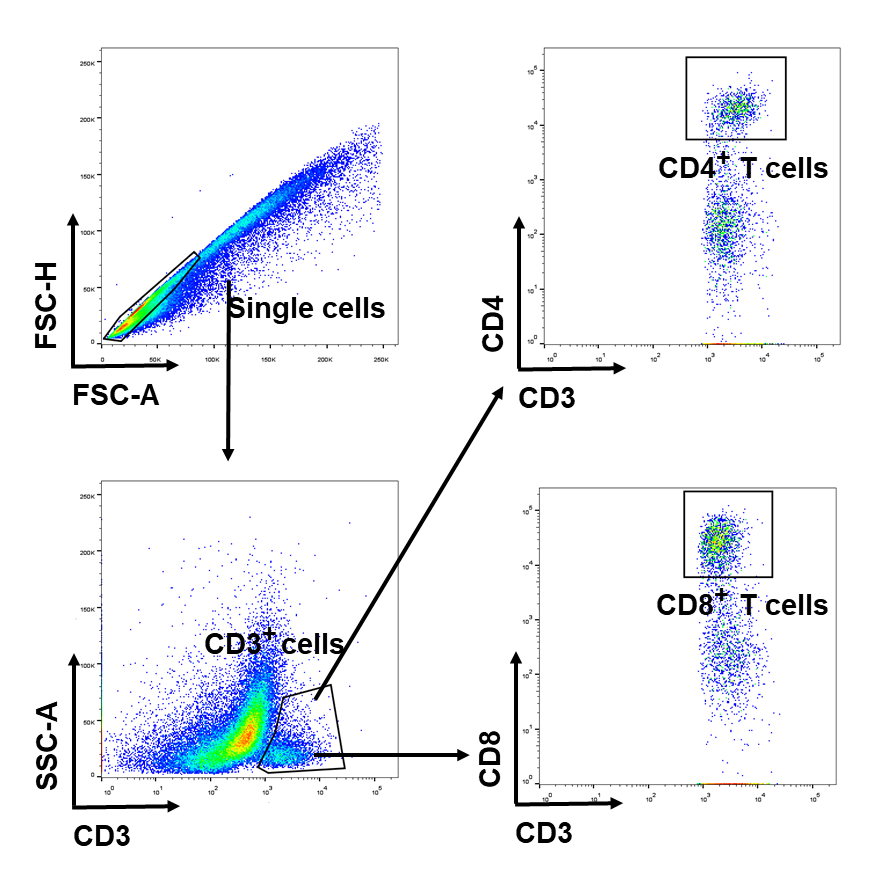


***Supplemental Figure 5*.**  Gating strategy for CD4**^+^** and CD8**^+^** T cells**.**


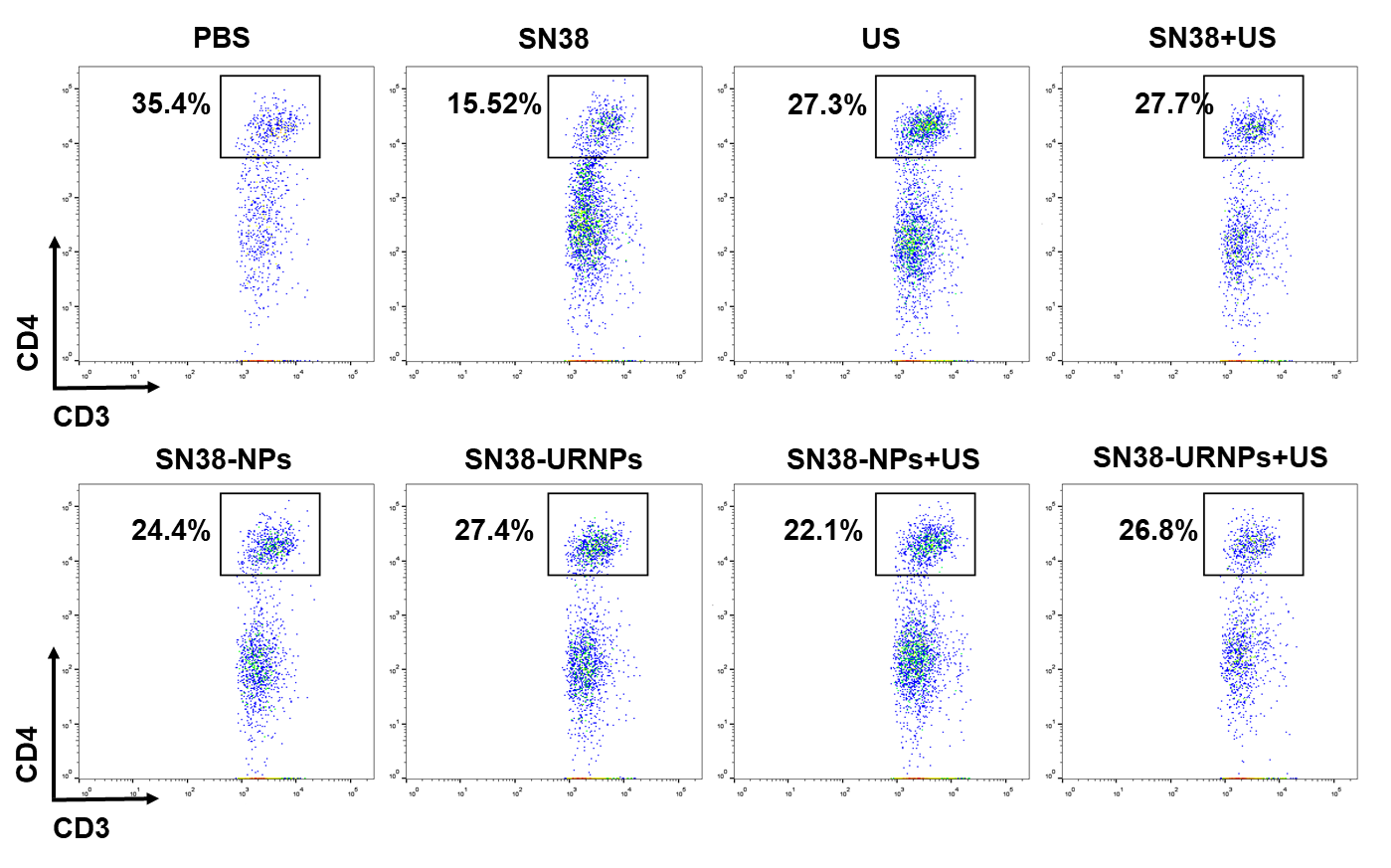


***Supplemental Figure 6*.** The representative ﬂow cytometric quantification of intertumoral infiltrating CD4**^+^** T.


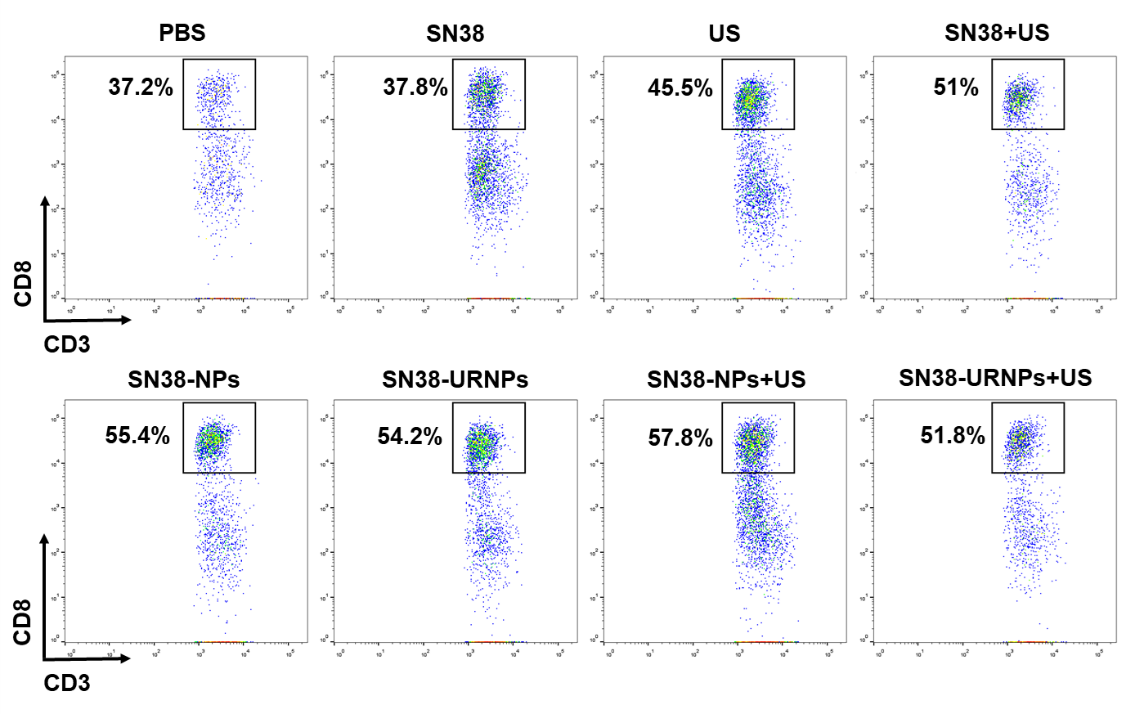


***Supplemental Figure 7*.** The representative ﬂow cytometric quantification of intertumoral infiltrating CD8**^+^** T.
